# *Toxoplasma gondii* Triggers Phosphorylation and Nuclear Translocation of Dendritic Cell STAT1 while Simultaneously Blocking IFNγ-Induced STAT1 Transcriptional Activity

**DOI:** 10.1371/journal.pone.0060215

**Published:** 2013-03-20

**Authors:** Anne G. Schneider, Delbert S. Abi Abdallah, Barbara A. Butcher, Eric Y. Denkers

**Affiliations:** Department of Microbiology and Immunology, College of Veterinary Medicine, Cornell University, Ithaca, New York, United States of America; Centre National de la Recherche Scientifique, France

## Abstract

The protozoan *Toxoplasma gondii* actively modulates cytokine-induced JAK/STAT signaling pathways to facilitate survival within the host, including blocking IFNγ-mediated STAT1-dependent proinflammatory gene expression. We sought to further characterize inhibition of STAT1 signaling in infected murine dendritic cells (DC) because this cell type has not previously been examined, yet is known to serve as an early target of in vivo infection. Unexpectedly, we discovered that *T. gondii* infection alone induced sustained STAT1 phosphorylation and nuclear translocation in DC in a parasite strain-independent manner. Maintenance of STAT1 phosphorylation required active invasion but intracellular parasite replication was dispensable. The parasite rhoptry protein ROP16, recently shown to mediate STAT3 and STAT6 phosphorylation, was not required for STAT1 phosphorylation. In combination with IFNγ, *T. gondii* induced synergistic STAT1 phosphorylation and binding of aberrant STAT1-containing complexes to IFNγ consensus sequence oligonucleotides. Despite these findings, parasite infection blocked STAT1 binding to the native promoters of the IFNγ-inducible genes *Irf-1* and *Lrg47*, along with subsequent gene expression. These results reinforce the importance of parasite-mediated blockade of IFNγ responses in dendritic cells, while simultaneously showing that *T. gondii* alone induces STAT1 phosphorylation.

## Introduction

*Toxoplasma gondii* is among the most successful parasitic microorganisms, infecting virtually all warm-blooded animals. Up to one-third of the human population worldwide is infected with this protozoan [1]. Most infections are asymptomatic or present with mild flu-like symptoms and the parasite establishes life-long infection characterized by presence of latent cysts in host tissues. Should immunocompromise develop, as in organ transplant recipients and AIDs patients, cyst reactivation can occur and lead to deadly encephalitis [2]. Fetuses can also become infected *in utero* during primary maternal infection with devastating consequences including blindness, mental retardation, and death [3]. Three main clonal lineages of the parasite have been identified in humans and domestic animals in North America, which differ in terms of virulence in mice [4]. Type I strains are the most virulent (LD50 = 1), resulting in death of the mouse during acute infection, whereas types II and III strains are much less virulent and capable of establishing chronic infection [5]. All three strain types can cause disease in humans, although type I strains may be more common in cases of ocular disease [6]. Recently, a fourth clonal lineage has been identified in wildlife populations of North America [7], [8].

Cell-mediated immunity is crucial to limiting severity of *Toxoplasma* infection. Widely considered the major mediator of host resistance is the cytokine interferon-gamma (IFNγ), produced primarily by T cells and NK cells. This cytokine is necessary for the control of intracellular parasite replication both *in vivo* and *in vitro*, in both mice and humans [9]–[14]. Among many effects, IFNγ activates antimicrobial effector mechanisms such as the immunity-related p47-GTPases that facilitate degradation of the parasitophorous vacuole [15]–[17].

Critical to the action of IFNγ in inducing antimicrobial effector mechanisms is the transcription factor signal transducer and activator of transcription 1 (STAT1) [18], [19]. Indeed, in the absence of STAT1, *Toxoplasma*-infected mice succumb rapidly to infection [20], [21]. In the classical signaling pathway, IFNγ binds its receptor and signals through a Janus kinase (JAK)/STAT1 cascade. Activated JAKs phosphorylate the IFNγ-receptor, permitting recruitment of STAT1. STAT1 is phosphorylated on a key tyrosine residue, Y701, and translocates as a dimer to the nucleus to initiate transcription, binding to gamma-activated sequence (GAS) elements in the promoters of responsive genes [22]-[24]. Phosphorylation at a key serine residue, S727, is thought to facilitate maximal STAT1 transcriptional activity [25]–[27]. One of the key early response genes regulated by STAT1 is interferon regulatory factor-1 (*Irf1*) [28], [29], itself a transcription factor, the absence of which also increases susceptibility to *Toxoplasma* infection [30].

Given the vital impact on parasite survival, it may not be surprising that *Toxoplasma* possesses mechanisms to counteract the IFNγ/STAT1 pathway. This has been demonstrated in a variety of cell types, including bone marrow-derived macrophages (BMDM), microglia, astroglia, the monocyte/macrophage RAW264.7 cell line, as well as human and murine fibroblasts. The parasite has been shown to block IFNγ-mediated upregulation of MHC class I and II molecules, class II transactivator (CIITA), inducible nitric oxide synthase, the chemokine monokine induced by IFNγ (MIG), interferon-inducible GTPase 1 (IIGP1) and IRF1 [31]–[39]. In addition, genome-wide microarray analyses in human fibroblasts and murine macrophages have described global inhibition of IFNγ-mediated gene expression in infected cells [40]–[42].

The molecular mechanism of inhibition remains unclear. Some discrepancy exists as to whether *Toxoplasma* targets STAT1 itself. One study performed in RAW264.7 cells at a high multiplicity of infection (MOI) concluded that infection blocked IFNγ-mediated STAT1 phosphorylation, likely via upregulation of suppressor of cytokine signaling-1 (SOCS1) [39], while another study implicated partial STAT1 dephosphorylation in the nuclei of infected human fibroblasts [40]. However, other studies found that STAT1 phosphorylation and nuclear translocation were unimpaired in infected cells, suggesting instead a block in IFNγ-mediated gene transcription [38], [41], [42]. The mechanism by which this occurs likely involves impaired recruitment of histone modifying enzymes such as BRG-1 to certain gene promoters, thereby rendering native chromatin inactive for transcription [41]. However, the particular parasite factors or signaling pathways involved in the inhibition remain unknown.

In our study, we sought to investigate the impact of *Toxoplasma* infection on the IFNγ/STAT1 pathway in primary bone marrow-derived murine dendritic cells (BMDC). Dendritic cells serve as an important early target of in vivo infection, playing a critical role in parasite dissemination throughout the host [43]–[45]. They also play a pivotal role in immune initiation [46], and their genetic deletion results in acute susceptibility to *T. gondii* [47], [48]. We unexpectedly discovered that *Toxoplasma* alone induces STAT1 phosphorylation and nuclear translocation in infected BMDC, regardless of strain type. In addition, we observed synergistic STAT1 phosphorylation when infected cells were treated with IFNγ. Electrophoretic mobility shift assays (EMSAs) revealed the presence of an aberrant GAS-binding complex that increased during the infection period in the presence of IFNγ. However, transcription of IFNγ/STAT1-responsive genes was impaired in infected cells. Binding of STAT1 to the native *Irf-1* promoter in response to IFNγ was also abrogated. These findings reveal that *Toxoplasma* triggers phosphorylation of STAT1 that nonetheless is unable to act as a transcription factor for typical IFN-γ-regulated genes.

## Results

### *Toxoplasma* alone induces rapid and sustained phosphorylation and nuclear translocation of STAT1 in BMDC

**Figure 1:**
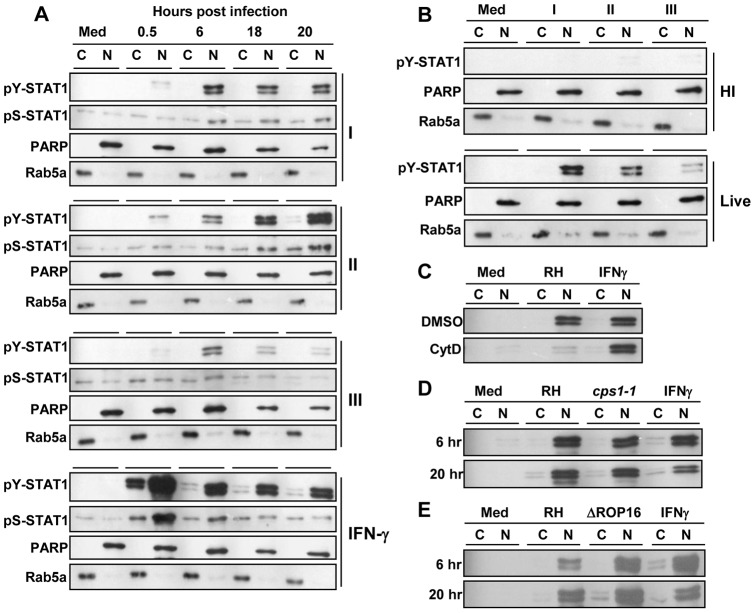
*Toxoplasma* induces STAT1 phosphorylation and nuclear translocation in BMDC. (A) BMDC were left in medium alone (Med), infected with type I (RH), II (PTG) or III (M774.1) strains of *Toxoplasma* (3∶1 ratio of parasites to cells), or treated with murine IFNγ (100 ng/ml), prior to fractionation into cytoplasmic (C) and nuclear (N) extracts at the time points indicated. Samples were subjected to immunoblot analysis for phospho-Tyr701-STAT1 (pY-STAT1) and phospho-Ser-STAT1 (pS-STAT1). PARP and Rab5a served as loading controls for nuclear and cytoplasmic fractions, respectively. (B) Cells were infected with live parasites of the three strains as in (A) or exposed to heat-inactivated (HI) tachyzoites for six hours. Cytoplasmic and nuclear fractions were collected and immunoblot analyis was performed as in (A). (C) RH parasites were pre-treated for 10 min on ice with 1 μM cytochalasin D (CytD) prior to infection in the continued presence of the drug. Cells treated with the solvent DMSO alone served as controls. Cells were fractionated after six hours and subjected to immunoblot analysis for pY-STAT1. (D and E) BMDC were treated with IFNγ or infected with RH in comparison with either the *cps1-1* replication-deficient strain (D) or the ΔROP16 strain (E). Samples were fractionated after 6 and 20 hours and subjected to immunoblot analysis for pY-STAT1. All experiments were repeated at least three times with similar results.

In order for STAT1 to be active as a transcription factor, it must be phosphorylated on a key tyrosine residue (Tyr701) prior to translocation to the nucleus [49]. To determine if parasites alone induce activation of STAT1, murine BMDC were infected with representative strains of the three primary clonal lineages of *Toxoplasma* (Type I – RH, Type II – PTG, Type III – M774.1), then fractionated into nuclear and post-nuclear extracts prior to immunoblot analysis with a phosphorylated STAT1-Tyr701 specific antibody (Fig. 1A). Cells treated with IFNγ (100 ng/ml) served as a positive control. As expected, BMDC responded to IFNγ treatment with robust STAT1 phosphorylation and nuclear translocation, peaking at the early 30 minute time point. Unexpectedly, infection with *Toxoplasma* alone also induced phosphorylation and nuclear translocation of STAT1, independent of strain type. (Fig. 1A). In contrast to IFNγ, *Toxoplasma*-mediated STAT1 phosphorylation was slower to develop, reaching peak levels at later timepoints. STAT1 phosphorylation declined over time with the type III strain M774.1 (Fig. 1A), and this correlated with poor survival of that particular strain. Infection with another type III strain (CTG) resulted in sustained STAT1 activation (data not shown). Phosphorylation of a key serine residue (Ser727) is thought to be required for maximal transcriptional activity of STAT1 [25]. Although not as strong as with IFNγ treatment, *Toxoplasma* infection was capable of inducing sustained STAT1 Ser727 phosphorylation (Fig. 1A). Therefore, *Toxoplasma* infection alone induces activation of STAT1 and mediates its translocation to the nucleus.

### STAT1 activation requires invasion by live parasites, and is independent of parasite replication and the rhoptry kinase ROP16

To further explore the mechanism by which parasites could trigger STAT1 phosphorylation, BMDC were infected with live parasites or treated with heat-inactivated tachyzoites prior to immunoblot analysis. As evident in Fig. 1B, heat-inactivated parasites were unable to initiate STAT1 phosphorylation. To confirm a requirement for active invasion, BMDC were infected with *Toxoplasma* in the presence of cytochalasin D, a drug that blocks actin polymerization and thereby also interferes with parasite gliding motility required for cell invasion [50]. STAT1 phosphorylation and nuclear translocation were largely absent in the presence of the drug, whereas the response to IFNγ stimulation was unaffected (Fig. 1C). Taken together, these results indicate a requirement for active invasion by live tachyzoites for parasite-mediated STAT1 activation.

Given the kinetics of parasite-induced STAT1 activation, with increasing levels of phosphorylated STAT1 detected at six hours and beyond, we next asked whether parasite replication could play a role. To address this question, a replication-deficient strain known as *cps1-1* was compared to the parental strain, RH, in terms of STAT1 activation. The *cps1-1* strain lacks an enzyme essential for uracil synthesis, and therefore parasite replication does not occur in uracil-free medium [51]. BMDC were infected with either RH or *cps1-1* in the absence of uracil prior to immunoblot analysis. There was no detectable difference in STAT1 phosphorylation between the two strains (Fig. 1D), indicating that replication of the parasite was not required.

The requirement for invasion by live parasites for STAT1 phosphorylation implicated a parasite-derived secretory kinase. The rhoptry kinase ROP16 is known to directly phosphorylate STAT3 and STAT6 [52], [53] resulting in inhibition of proinflammatory cytokine production and promotion of arginase-1-dependent growth control [54], [55]. We therefore asked whether STAT1 could be a collateral target of ROP16 by comparing BMDC infected with the parental RH strain with a ROP16-deleted strain (ΔROP16). As shown in Fig. 1E, robust STAT1 phosphorylation was maintained even in the absence of ROP16.

### STAT1 activation is confined to infected cells

**Figure 2:**
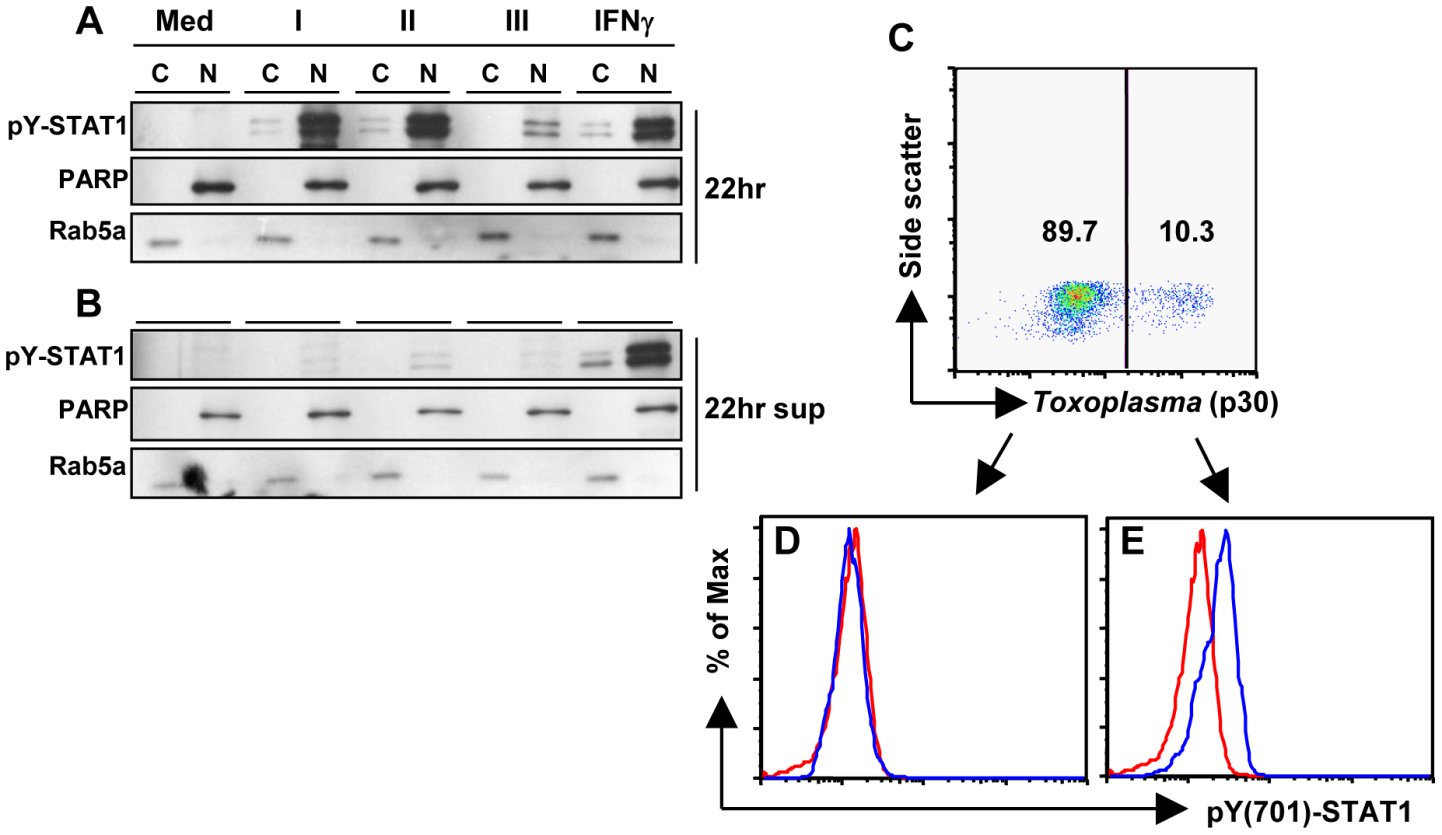
STAT1 phosphorylation is confined to infected cells. (A) BMDC were left in medium alone (Med), infected with type I (RH), type II (PTG) or type III (M774.1) strains of *Toxoplasma* (3∶1 ratio of parasites to cells) or treated with murine IFNγ (100 ng/ml). After 22 hours supernatants were collected (22 hr sup), centrifuged and filtered to remove debris and parasites, and subsequently transferred to additional untreated/uninfected BMDC for an additional 20 hours (B). For all samples (A and B), cytoplasmic (C) and nuclear (N) fractions were prepared and subjected to immunoblot analysis with phospho-Tyr701-STAT1 (pY-STAT1). PARP and Rab5a served as cytoplasmic and nuclear loading controls, respectively. (C – E) Cells were infected with the RH strain at a ratio of 0.5 parasites/cell for 20 hours, then subjected to intracellular staining for *Toxoplasma* (anti-p30/SAG-1) and pY-STAT1 prior to flow cytometric analysis. BMDC were gated on uninfected (D) and infected (E) populations to assess pY-STAT1 expression (blue lines) relative to staining with an isotype control antibody (red lines). All experiments were repeated at least twice with similar results.

We next asked whether the increasing amount of phosphorylated STAT1 over time occurred through secretion of a soluble DC factor in response to infection. To address this, supernatants from BMDC infected overnight were collected, centrifuged and filtered to remove parasites and cell debris, then transferred to uninfected BMDC. Cells treated with supernatants were compared with infected cells by immunoblot analysis of pY-STAT1. IFNγ treatment served as a positive control. Cells infected with each of the three parasite strains (I, II, III) or treated with IFNγ demonstrated the expected STAT1 phosphorylation response (Fig. 2A). Transferred supernatant containing IFNγ was also capable of inducing STAT1 activation, but supernatant from infected cells was not sufficient, regardless of strain type (Fig. 2B). From these results, we conclude that a soluble, secreted factor arising from infection is not involved in parasite-triggered STAT1 activation.

A closely related possibility was that parasite-initiated upregulation of a DC surface membrane-bound molecule might trigger STAT1 activation in bystander cells. To address this possibility, BMDC were infected with a low infection ratio of 0.5 parasites per cell overnight prior to flow cytometric analysis for pY-STAT1 expression (Fig. 2C–E). The cell population was first analyzed in terms of infection status (p30/SAG-1 positive, Fig. 2C), then gated into separate uninfected (Fig. 2D) and infected (Fig. 2E) subpopulations to compare pY-STAT1 expression (blue line) versus the isotype control (red line). Unlike the uninfected group (Fig. 2D), cells infected with RH showed a shift in pY-STAT1 expression relative to isotype control (Fig. 2E), confirming that STAT1 phosphorylation is confined to infected cells.

### Parasite-induced nuclear STAT1 binds IFN-γ-activated sequences (GAS) in vitro

**Figure 3:**
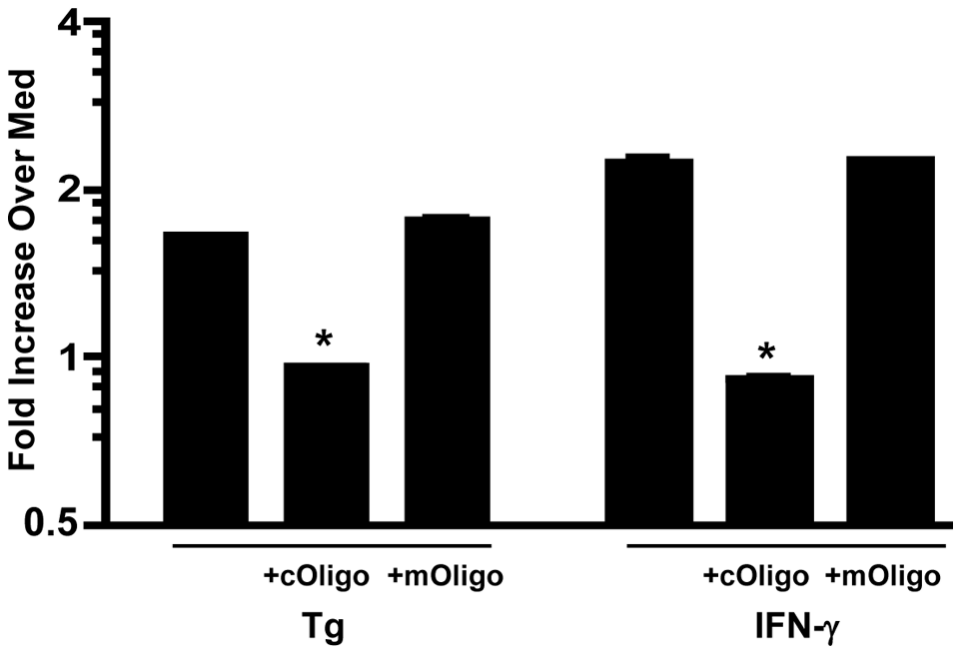
*Toxoplasma* induces *in vitro* STAT1 binding activity in BMDC. Nuclear extracts were prepared from cells infected with the RH parasite strain (Tg, *Toxoplasma gondii*; 3∶1 parasites to cells) or treated with IFNγ (100 ng/ml) for 6 hours. The *in vitro* binding activity of nuclear STAT1 to solid phase IFNγ-activated sequence (GAS) oligonucleotides was assessed using an ELISA-based method. Binding activity is expressed as fold increase over cells cultured in medium alone (Med, value of 1). +cOligo, addition of soluble competitive oligonucleotides; +mOligo, addition of mutated non-competitive oligonucleotides. The experiment was repeated three times with similar results. *, p<0.05 comparing nuclear extracts alone with nuclear extracts plus cOligo.

Since *Toxoplasma* infection triggered STAT1 phosphorylation and nuclear translocation, we asked whether the activated STAT1 was indeed functional. When IFNγ-activated STAT1 homodimers translocate to the nucleus, they recognize and bind a palindromic IFNγ-activated consensus sequence (GAS) in the promoters of responsive genes in order to initiate transcription [56]. To assess STAT1 binding, a transcription factor binding assay was performed whereby nuclear extracts from IFNγ-treated or parasite-infected BMDC were incubated with plate-immobilized STAT1 consensus sequences. Binding activity was subsequently detected using an anti-STAT1 antibody and quantified using an ELISA-based method. As shown in Fig. 3, treatment with the positive control IFNγ or infection with the parasite strain RH (Tg) induced significant STAT1 binding activity. Addition of a competitive oligonucleotide (cOligo) reduced STAT1 binding to the medium control activity (set to 1), establishing binding specificity. Addition of a mutated oligo (mOligo) that could not compete for binding restored STAT1 binding activity to initial values in both cases. We conclude that *Toxoplasma*-triggered nuclear STAT1 is capable of recognizing and binding IFNγ-responsive consensus sequences.

### *Toxoplasma* and IFNγ together result in synergistic STAT1 phosphorylation and consensus sequence binding

**Figure 4:**
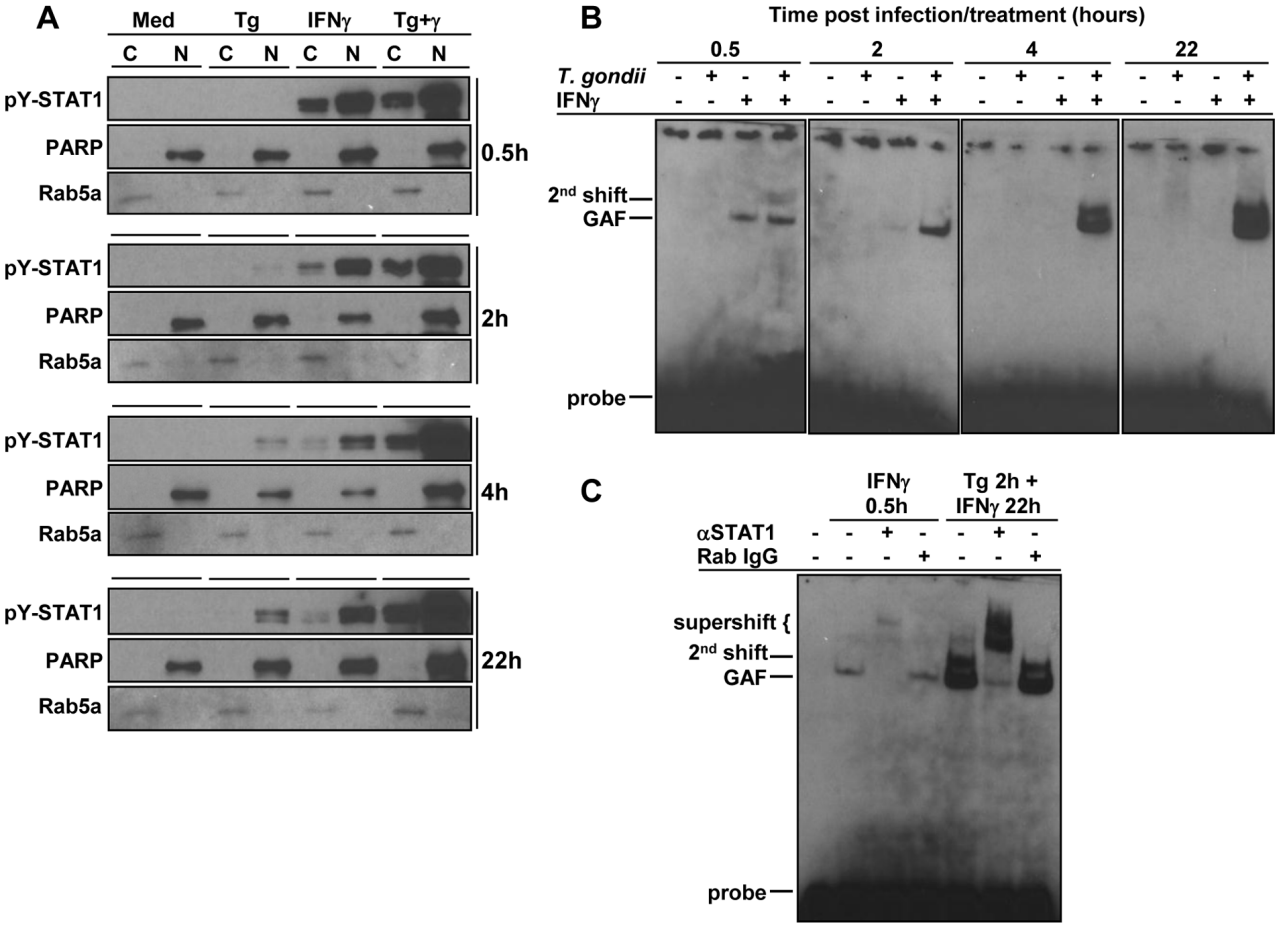
*Toxoplasma* synergizes with IFNγ in terms of phosphorylation and in vitro binding activity of STAT1. BMDC were left in medium alone (Med), infected with *Toxoplasma* (Tg, RH strain) at a ratio of 3 parasites per cell, treated with IFNγ (100 ng/ml), or infected with *Toxoplasma* followed 2 hours later by IFNγ treatment for the indicated times. In (A), cells were fractionated into cytosolic (C) and nuclear (N) extracts prior to immunoblot analysis for phospho-Tyr701-STAT1 (pY-STAT1). PARP and Rab5a served as loading controls for nuclear and cytoplasmic fractions, respectively. In (B) and (C), nuclear extracts were tested for binding to biotinylated probes containing the gamma-activated sequence (GAS) from the *Irf-1* promoter by EMSA. Supershift assays were also performed with samples in (C) using an antibody against STAT1α or normal rabbit IgG as a negative control. Experiments were repeated at least three times with similar results. GAF, gamma-activated factor (STAT1 homodimer).

The ability of *Toxoplasma* to induce STAT1 binding to consensus sequences was surprising, given that others have shown that the parasite blocks IFNγ-mediated STAT1-dependent activity [38], [39], [41], [42]. To determine what impact the parasite may have on IFNγ-induced STAT1 phosphorylation, BMDC were pre-infected with *Toxoplasma* for two hours followed by subsequent IFNγ treatment. Cells were infected with *Toxoplasma* or treated with IFNγ alone for comparison, and analyzed by immunoblotting for pY-STAT1. *Toxoplasma* alone or IFNγ treatment alone triggered STAT1 phosphorylation and nuclear translocation as expected (Fig. 4A). To our surprise, *Toxoplasma* and IFN-γ together provided a synergistic signal resulting in greatly enhanced STAT1 phosphorylation. Synergistic STAT1 phosphorylation levels were maintained and even increased with IFNγ treatment over time (Fig. 4A).

We next asked whether pre-infection could also enhance IFNγ-induced STAT1 binding to labeled gamma-activated sequence (GAS) oligonucleotides from the *Irf-1* promoter in an electrophoretic mobility shift assay (EMSA). Upon binding of its receptor, IFNγ triggers formation of a phosphorylated STAT1 homodimer known as gamma-activated factor (GAF) that is capable of binding to GAS elements in responsive promoters [49]. As expected, 30 minutes of IFNγ stimulation resulted in strong binding to the labeled GAS oligonucleotides (Fig. 4B). Addition of anti-STAT1 antibody but not normal rabbit IgG resulted in a supershift, indicating that the single complex contained STAT1, consistent with GAF (Fig. 4C). When *Toxoplasma* alone was added to cells, no consistent shifts were detected (Fig. 4B), although this is likely due to lack of assay sensitivity because oligonucleotide binding was observed in the transcription factor ELISA (Fig. 3). Previous studies have indicated that *Toxoplasma* pre-infection reduces GAF formation in IFNγ-treated cells [32], [41]. However, when BMDC were pre-infected for two hours and subsequently treated with IFNγ for 30 minutes, no reduction in GAF was observed (Fig. 4B). Additionally, synergistic GAF formation was evident following IFNγ treatment for 2, 4 or 22 hours (Fig. 4B). Interestingly, *Toxoplasma* in combination with IFNγ also induced an aberrant complex with lower electrophoretic mobility than GAF. We further explored this through a time course, noting that formation of this second complex increased over time, peaking at 22 hours (Fig. 4B). Supershift experiments confirmed that the aberrant complex also contained STAT1 (Fig. 4C). Taken together, these data demonstrate that *Toxoplasma* pre-infection of BMDC leads to synergistic activation and binding of atypical STAT1 complexes to GAS sequences in response to IFNγ.

### *Toxoplasma* blocks IFNγ-induced, STAT1-dependent responses despite STAT1 activation

**Figure 5:**
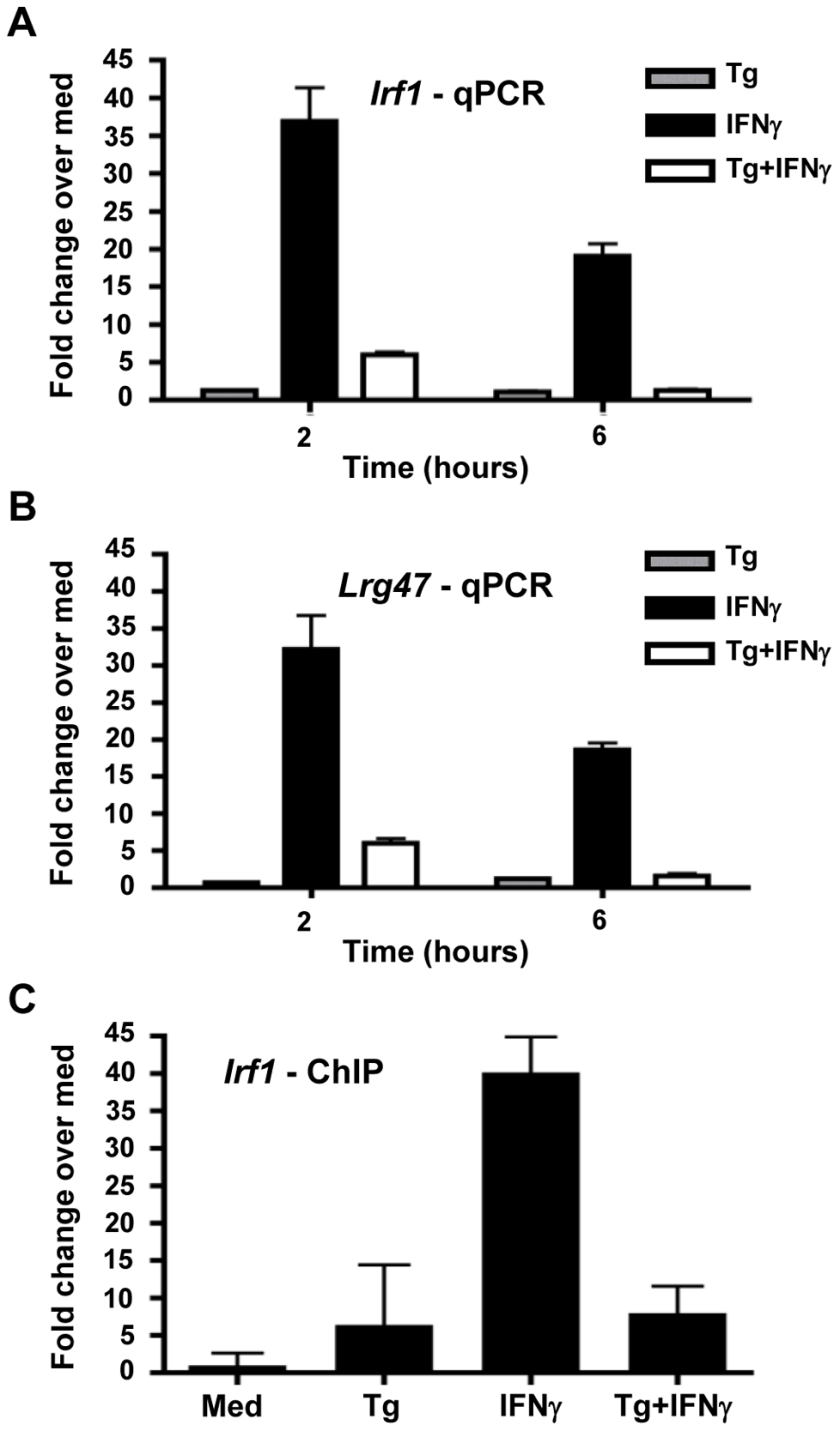
*Toxoplasma* blocks IFNγ-driven STAT1-dependent gene induction. BMDC were infected with the RH strain of *T. gondii* (Tg, ratio of 3 parasites/cell), treated with treated IFNγ (100 ng/ml), or pre-infected for 2 hours with RH prior to addition of IFNγ (Tg+IFNγ). At the indicated time points post cytokine treatment, total RNA was harvested and reverse-transcribed to cDNA prior to qPCR amplification of the IFNγ-responsive, STAT1-dependent genes *Irf-1* (A) and *Lrg-47* (B). Fold change in gene expression is expressed relative to BMDC in medium alone. Samples were normalized to the house-keeping gene GAPDH. To assess native chromatin binding, ChIP was performed using an anti-STAT1α antibody followed by qPCR amplification with primers specific for the *Irf-1* promoter (C). Experimental conditions are replicated as in (A and B), with a time point of 2 hours shown. In (C), fold change in promoter binding is expressed relative to untreated cells (Med). Samples were normalized to input chromatin. Experiments were repeated at least three times with similar results.

Given the evidence of STAT1 activation and binding in *Toxoplasma*-infected BMDC, we hypothesized that in this cell type, tachyzoites may promote IFNγ/STAT1-dependent gene expression, even though in other cell types the parasite has the opposite effect. To address this, we utilized real-time quantitative PCR to examine the expression of known STAT1-dependent genes triggered by IFNγ stimulation, including the transcription factor interferon regulatory factor 1 (*Irf1)* [28], [29], and members of the p47 GTPase family known to be essential for survival during *in vivo* infection, *Lrg-47* and *Igtp* [17], [20], [57], [58]. As expected, treatment with IFNγ resulted in induction of both *Irf1* (Fig. 5A) and *Lrg-47* (Fig. 5B), with peak fold induction over untreated samples at two hours post treatment. However, despite evidence of activated STAT1 in infected cells, *Toxoplasma* infection alone did not result in induction of the same genes (Fig. 5A and B). Furthermore, when cells were first pre-infected for two hours followed by IFNγ stimulation, expression of the IFNγ-inducible target genes was blocked by presence of the parasite (Fig. 5A and B). Expression of the p47 GTPase *Igtp* was also examined with similar results (data not shown).

To further explore the mechanism behind the parasite-mediated block in gene expression, we next asked whether activated STAT1 present in the nucleus could bind to the native promoter of an IFNγ responsive gene. *Toxoplasma* is known for the ability to interfere with permissive chromatin remodeling and thereby prevent binding of transcription factors to various promoters [41], [59], [60]. To address this possibility in BMDC, we performed chromatin immunoprecipitation (ChIP) assays using an anti-STAT1 antibody. Precipitated DNA was subsequently amplified by quantitative PCR using primers specific for the *Irf1* promoter. As anticipated, IFNγ induced strong STAT1 binding to the native *Irf1* promoter (Fig. 5C). In contrast, pre-infection with *Toxoplasma* resulted in blockage of IFNγ-mediated binding while infection alone resulted in little to no binding (Fig. 5C). Taken together with the gene expression results (Fig. 5A and B), these data indicate that although activated STAT1 can bind target oligonucleotides (Fig. 3, Fig. 4B and C) and translocate into the nucleus of infected cells (Fig. 1), transcriptional activity at STAT1-dependent, IFNγ-responsive genes remains blocked by the parasite. Therefore, our data reinforce the importance of an IFNγ-transcriptional blockade as an immune evasion mechanism in dendritic cells. We also demonstrate that the parasite itself triggers phosphorylation and nuclear translocation of STAT1. The function of STAT1 in the latter context remains to be determined.

## Discussion

Subversion of host immune responses, particularly those induced by the IFNγ/STAT1 signaling cascade, is now recognized as a key feature that contributes to the success of *Toxoplasma* as a parasitic organism. Although IFNγ is required for host defense, the response must also be partially counteracted to allow persistent life-long infection. Blockade of IFNγ-mediated transcription in infected cells as a means of immune evasion has been previously demonstrated in a range of cell types. In our study, we further highlight the prime importance of immune evasion in dendritic cells that are strategically situated at the front line of the host response to infection.

A key signaling intermediate downstream of IFNγ in anti-parasite immune responses is the transcription factor, STAT1. We initially anticipated that *Toxoplasma* would interfere with STAT1 activity in BMDC, either during the phosphorylation process as previously demonstrated in RAW264.7 cells [39] or by blocking downstream transcription as supported by others [36], [38], [40]–[42]. Therefore, we were surprised to discover that *Toxoplasma* itself induces STAT1 phosphorylation and nuclear translocation. Concurrent with our work, another group recently published similar findings in human fibroblasts, confirming our results [42]. We considered the possibility that STAT1 could be activated by the parasite rhoptry kinase ROP16, since that kinase is already known to phosphorylate STAT3 and STAT6 [52], [53]. However, in contrast to the other group's findings [42], we found that parasite-induced STAT1 phosphorylation is not dependent on the rhoptry kinase ROP16 because we observed STAT1 activation in response to a Type II strain, which possesses an inactive ROP16 allele. Furthermore, a Type I strain in which ROP16 was genetically deleted retained STAT1 activation capability. The reason for this discrepancy is unclear, but could be due to the species and cell type differences between studies. Nevertheless, we conclude that *Toxoplasma* induces ROP16-independent STAT1 phosphorylation and nuclear translocation in BMDC.

We do not yet know how STAT1 is activated by the parasite. However, we note that although ROP16 plays a role in maintaining STAT3 activation in infected cells, there is a substantial ROP16-independent STAT3 phosphorylation response during early infection [54]. We considered whether this unknown ROP16-independent pathway could also be responsible for parasite-induced STAT1 phosphorylation. However, this scenario seems unlikely given that robust ROP16-independent STAT3 phosphorylation occurs very rapidly (within the first 15 minutes) whereas parasite-induced STAT1 phosphorylation increases gradually over time. In terms of STAT1 phosphorylation, active invasion by parasites is necessary. Yet, in the presence of cytochalasin D, a drug that prevents invasion while allowing discharge of rhoptries but not dense granules [61], STAT1 activation is prevented. This raises the possibility that a dense granule protein might activate STAT1, particularly insofar as dense granule protein GRA15 has recently been implicated in NFκB activation in the host cell [62]. We note in that study activation of NFκB by GRA15 occurs with delayed kinetics, requiring ∼4 hours after infection to achieve substantial p65 nuclear accumulation. The kinetics correlate with those we see during *Toxoplasma*-mediated STAT1 activation. We do not anticipate that GRA15 itself targets STAT1, given that only type II parasites express the active protein whereas we see strain-independent STAT1 phosphorylation, but nonetheless another dense granule protein could be responsible.

Interestingly, the combination of *T. gondii* and IFNγ synergized to stimulate potent STAT1 activation and nuclear translocation. It is not clear whether synergistic phosphorylation is mediated via cross-talk of parasite and IFNγ-induced signaling pathways, or rather whether failure to dephosphorylate activated STAT1 could be the cause. It is possible that parasite infection down-regulates the IFNγ-induced expression of phosphatases such as suppressor of cytokine signaling 1 (SOCS1), known to negatively regulate STAT1 signaling. In this regard, IFNγ-mediated SOCS1 expression has been shown to be repressed by the parasite [40], [41]. However, other data suggest that *Toxoplasma* induces SOCS1 expression in a ROP16-dependent manner [39], [55]. Clearly, further work is required to determine the biologically relevant role, if any, of *Toxoplasma*-mediated interference of phosphatase activity.

In addition to synergistic STAT1 phosphorylation, we observed formation of an aberrant STAT1-containing complex, capable of binding to GAS oligonucleotides in vitro. A similar complex was noted by Lang et al in murine macrophages [41]. In that study, *Toxoplasma* reduced IFNγ-mediated STAT1 homodimer formation by inducing formation of the aberrant complex. We did not observe a similar reduction and in fact both complexes increased over the 24 hour time course of infection. We also did not observe GAF formation in response to IFNγ at time points beyond 30 minutes by EMSA despite still being able to detect phosphorylated STAT1 in the nucleus by immunoblot analysis. We attribute this to a difference in assay sensitivity, given that IFNγ-mediated STAT1 phosphorylation is substantially reduced at later time points as part of a negative feedback response (Fig. 1A and 4A). Assay sensitivity differences may also explain why we could detect an ∼2-fold increase in oligonucleotide binding in response to IFNγ after 6 hours by the ELISA-based method (Fig. 3) that was not seen later on with the EMSA method at a similar time point (Fig. 4B). Regardless, an aberrant STAT1-containing complex did accumulate substantially over time in the parasite plus IFNγ-treated group. Thus, it is possible that the additional parasite or host proteins involved may play a role in the mechanism of inhibition of IFNγ/STAT1-dependent gene transcription. Such a protein could allow binding of STAT1 to oligonucleotide sequences *in vitro*, but alter interactions with native chromatin and/or chromatin modifiers to prevent *in vivo* transcriptional activity of STAT1.

Our data corroborate previous studies with other cell types showing that *Toxoplasma* blocks the transcription of IFNγ-responsive, STAT1-dependent genes. This occurs despite the parasite's ability to activate STAT1. It is possible that *Toxoplasma*-activated STAT1 retains function in the regulation of a unique subset of genes, distinct from IFNγ-responsive, STAT1-dependent genes such as *Irf1* and the p47-GTPases. Indeed, a microarray study performed in bone marrow-derived macrophages identified a subset of IFNγ-responsive genes that were up-regulated by the presence of the parasite [41]. That study was not performed in STAT1 null macrophages to determine whether STAT1 was required for the increase in transcription. It would be of interest in the future to perform microarray studies in infected BMDC in the presence and absence of STAT1 to determine what role, if any, *Toxoplasma*-activated STAT1 plays in the host response to infection.

## Materials and Methods

### Ethics Statement

All experiments with animals in this study were performed in strict accordance with the recommendations in the Guide for the Care and Use of Laboratory Animals of the National Institutes of Health. The protocols were approved by the Institutional Animal Care and Use Committee at Cornell University (Permit Number: 1995–0057). All efforts were made to minimize animal suffering.

### Mice and Parasites

Female C57BL/6 mice of 6–8 weeks of age were purchased from either the Jackson Laboratory or Taconic Farms. All mice were housed under specific pathogen-free conditions at the Transgenic Mouse Core Facility at Cornell University's College of Veterinary Medicine, which is accredited by the Association for the Assessment and Accreditation of Laboratory Animal Care. Tachyzoites of the *Toxoplasma* strains RH (type I), PTG (type II), M774.1 (type III), *cps1-1* (type I attenuated strain), and ΔROP16 (RH/type I background) were maintained by biweekly passage on human foreskin fibroblast monolayers (American Type Tissue Collection) in DMEM (Life Technologies) supplemented with 1% bovine growth serum (Hyclone), 100 U/ml penicillin (Life Technologies), and 0.1 mg/ml streptomycin (Life Technologies). The RH parasites deficient in the rhoptry kinase ROP16 (ΔROP16) were generated as described previously [54] and were kindly provided by D. Bzik and B. Fox (Dartmouth Medical Center). The attenuated uracil auxotroph strain *cps1-1* [51] was supplemented with 250 nM uracil during passage to permit replication. Parasite cultures were tested every 6–8 weeks for *Mycoplasma* contamination using a commercial PCR-ELISA based kit (Roche Applied Systems).

### Bone Marrow-Derived Dendritic Cell (BMDC) Culture

Bone marrow was flushed from the femur and tibia of a C57BL/6 mouse and prepared as a single cell suspension in BMDC medium composed of RPMI 1640 (Fisher Scientific) supplemented with 10% fetal calf serum (Hyclone), 100 U/ml penicillin (Life Technologies), 0.1 mg/ml streptomycin (Life Technologies), 50 μM 2-mercaptoethanol (Sigma) and 20 ng/ml GM-CSF (Peprotech). Cells were plated on 100×15 mm standard sterile polystyrene Petri dishes (Fisher Scientific) and cultured for 9 days at 37°C in 5% CO_2_. Fresh BMDC medium was added on day 3. On day 6, fresh BMDC medium containing 50 mM 2-mercaptoethanol was added. On day 8, 200 ng of GM-CSF was added per plate. On day 9, non-adherent cells (BMDC) were collected and resuspended in DMEM (Life Technologies) supplemented with 10% bovine growth serum (Hyclone), 50 mM 2-mercaptoethanol (Sigma) and the following reagents from Life Technologies: 100 U/mL penicillin (Life Technologies), 0.1 mg/ml streptomycin (Life Technologies), 0.1 mM non-essential amino acids, 1 mM sodium pyruvate, and 3% HEPES (1M).

### *In vitro* Infections and Stimuli

Infection of BMDCs was accomplished through addition of tachyzoites to cell cultures at a ratio of 3∶1 (parasites:BMDCs) followed by brief centrifugation (200×g, 3 min) to initiate contact between cells and parasites. In other experiments, cells were treated with recombinant murine IFNγ (100 ng/mL, Peprotech) or first pre-infected with tachyzoites for two hours followed by IFNγ treatment. For cytochalasin D experiments, BMDC were pretreated for 10 min at 4°C with cytochalasin D (Calbiochem) at a final concentration of 1 μM or with the solvent DMSO (Sigma) alone. Cells were then infected with tachyzoites or treated with recombinant murine IFNγ for 6 hours in the continued presence of the drug. For the supernatant transfer experiments, supernatants from cells infected with tachyzoites or treated with IFNγ overnight were collected, centrifuged to remove cellular debris, and filtered through 0.2 μM filters (Corning) prior to addition to untreated cells.

### Immunoblot Analysis

The following primary antibodies from Cell Signaling were used in immunoblotting studies: anti-phospho-STAT1-Tyr 701 (catalog no. 9167), anti-phospho-STAT1-Ser727 (catalog no. 9177), and anti-PARP (catalog no. 9542). The Rab5a antibody (catalog no. sc-309) was obtained from Santa Cruz Biotechnology. Cells (3×10^6^/sample) were fractionated into cytoplasmic and nuclear fractions using a nuclear extract kit (Active Motif) as directed. Samples were subsequently diluted with 2x reducing SDS sample buffer. After 5 min at 100°C, samples were separated by 10% SDS-PAGE and proteins were subsequently electrotransferred onto nitrocellulose membranes (Whatman). The membranes were blocked for one hour at room temperature in Tris-buffered saline containing 0.1% Tween 20, pH 7.6, (TBST) and 5% nonfat dry milk prior to the addition of primary antibody overnight at 4°C in TBST containing 5% bovine serum albumin (BSA) (Calbiochem). Membranes were subsequently washed with TBST prior to detection of primary antibody binding with a horseradish peroxidase-conjugated anti-rabbit IgG (Jackson ImmunoResearch, catalog no. 111-035-144) in TBST containing 5% nonfat dry milk for one hour at room temperature. Membranes were washed in TBST prior to visualization of bands using a chemiluminescence detection system (Thermo Scientific).

### Flow Cytometry

BMDC were infected with the type 1 parasite strain RH at a ratio of 0.5 parasites per cell and harvested for flow cytometric analysis after 20 hours. Samples were fixed for 10 minutes at room temperature with 3% formaldehyde, followed by permeabilization for 30 minutes at 4°C with ice-cold 100% methanol. Cells were plated at 2×10^6^ per well in a 96-well plate and washed twice with FACS buffer (1% BSA in PBS). Cells were stained with anti-phospho-STAT1-Tyr701 (Cell Signaling, catalog no. 9167) diluted in FACS buffer for 1 hour at room temperature. Samples were washed twice with FACS buffer prior to addition of an antibody mixture for 30 minutes at room temperature containing goat-anti-rabbit-Alexa Fluor 647 (Life Technologies, catalog no. A21245) to detect the primary p-STAT1 antibody, and anti-p30-FITC (Argene, catalog no. 12–132) to detect *Toxoplasma*-infected cells. Samples were again washed with FACS buffer prior to analysis on a FACS Calibur flow cytometer (BD Biosciences). Data were subsequently analyzed using FlowJo software (Tree Star).

### Transcription Factor DNA-binding ELISA

The presence of activated STAT1 complexes capable of binding to consensus oligonucleotides was assessed in BMDC nuclear extracts using the TransAM STAT Family Transcription Factor Assay Kit (Active Motif), per manufacturer's instructions. BMDC were either infected with the RH strain of parasites (ratio of 3 parasites per cell) or treated with recombinant murine IFNγ (Peprotech) for six hours prior to analysis. The STAT consensus nucleotide coated on the 96-well plates consisted of the following sequence: 5′-TTCCCGGAA-3′.

### Electrophoretic Mobility Shift Assay (EMSA)

To further confirm STAT1 binding-activity in nuclear extracts, EMSA was performed using a LightShift Chemiluminescent EMSA kit, per manufacturer's instructions (Thermo Scientific). Briefly, complementary oligonucleotides (5′-CATTTCGGGGAAA

TCGATC-3′ and 5′-GATCGATTTCCCCGAAATG-3′; IDT Technologies) designed against the GAS sequence of the *Irf-1* promoter (Ng et al, 2011) were biotinylated at the 5′ end prior to annealing equimolar quantities of each to create a labeled probe. Equal volumes of nuclear extract (from 3×10^6^ cells) were then incubated for 20 minutes with 200 fmol of biotinylated probe, 1 μg poly(I)-poly(C), binding buffer (Thermo Scientific), 2.5% glycerol, 50 mM KCl, 1 mM DTT, and 1 mM EDTA. Supershift assays were performed by the addition of 2 μg of supershift grade rabbit anti-STAT1α p91 (Santa Cruz Biotechnology, sc-591x) or normal rabbit IgG (Santa Cruz Biotechnology, sc-2027) for 20 minutes on ice prior to the addition of nuclear extract at room temperature for an additional 20 minutes. The protein-DNA complexes were resolved on a native 5% polyacrylamide gel (Bio-Rad) in 0.5x TBE buffer prior to transfer to a positively charged nylon membrane (Bio-Rad). Transferred DNA was crosslinked to the membrane using a commercial UV light crosslinking instrument (UV Stratalinker 2400, Stratagene) at 120 mJ/cm^2^ using the auto crosslink function. Shifts of the biotinylated DNA probe were detected using a streptavidin-horseradish peroxidase conjugate and chemiluminescent substrate (Thermo Scientific).

### Quantitative Reverse Transcriptase PCR

BMDC (3×10^6^) were treated with IFNγ and/or infected with the RH parasite strain prior to isolation of total RNA using the E.Z.N.A Total RNA Miniprep kit (Omega Bio-Tek). Samples were treated on column with DNase I (Agilent Technologies) during the total RNA isolation. cDNA was synthesized from total RNA using qScript cDNA SuperMix (Quanta Biosciences). Quantitative PCR was performed on cDNA samples using the SYBR green method (Quanta Biosciences) and the ABI Prism 7500 sequence detection system (Applied Biosystems). Expression of target genes was normalized to the housekeeping gene GAPDH, and the relative expression of treated samples versus an untreated control sample was calculated using the ΔC_T_ method. The primer sequences employed were: *Irf1* forward: 5′-TTGGCATCATGGTGGCTGT-3′; *Irf1* reverse: 5′-AAGGAGGATGGTCCCCTGTTT-3′; *Lrg47* forward: 5′-GAGACTGTGGCAACATTG

TCCC-3′, *Lrg47* reverse: 5′-CCGATGACTCGAAGTGCATTG-3′; *GAPDH* forward: 5′-AATGGTGAAGGTCGGTGTG-3′, *GAPDH* reverse: 5′-GTGGAGTCATACTGGAA.

CATGTA-3′; *Irf1* promoter forward: 5′-AGCTCTACAACAGCCTGATTTCCC-3′, *Irf1* promoter reverse: 5′-GCGCCGCGAAGAAATCTAAACACT-3′.

### Chromatin Immunoprecipitation (ChIP)

An anti-total-STAT1α antibody (ChIP grade, Santa Cruz Biotechnology, sc-591x) was used to precipitate STAT1-bound chromatin fragments. Normal rabbit IgG (Santa Cruz Biotechnology, sc-2027) was used as a negative control antibody. Assays were performed using the Magna ChIP G Chromatin Immunoprecipitation kit (Millipore, catalog no. 17–611) according to the manufacturer's instructions. Briefly, BMDC (10^7^/sample) were collected and fixed in 1% formaldehyde for 5 minutes at room temperature. Fixation was stopped by addition of glycine to the mixture. Samples were washed with cold PBS and resuspended in cell lysis buffer (Millipore) supplemented with protease inhibitors for 15 minutes at 4°C. Resulting nuclear pellets were spun down and resuspended in nuclear lysis buffer (Millipore) prior to chromatin shearing by sonication in an ice bath with twelve, thirty second pulses at high power using the Bioruptor (Diagenode). Immunoprecipitation of the sheared chromatin was carried out overnight at 4°C via addition of dilution buffer (Millipore), antibody (2 μg), and protein G magnetic beads. Beads were washed, protein/DNA crosslinks were reversed, proteins were digested with Proteinase K, and immunoprecipitated DNA was eluted per manufacturer's instructions. The retrieved DNA was then subjected to amplification by quantitative real-time PCR using promoter-specific primers. Immunoprecipitated samples were normalized to respective input controls (10% of input sheared chromatin).

### Statistical Analysis

Statistically significant differences between groups were assessed using the unpaired Student's T-test. Values for p<0.05 were considered significant. All experiments were performed a minimum of three times.
